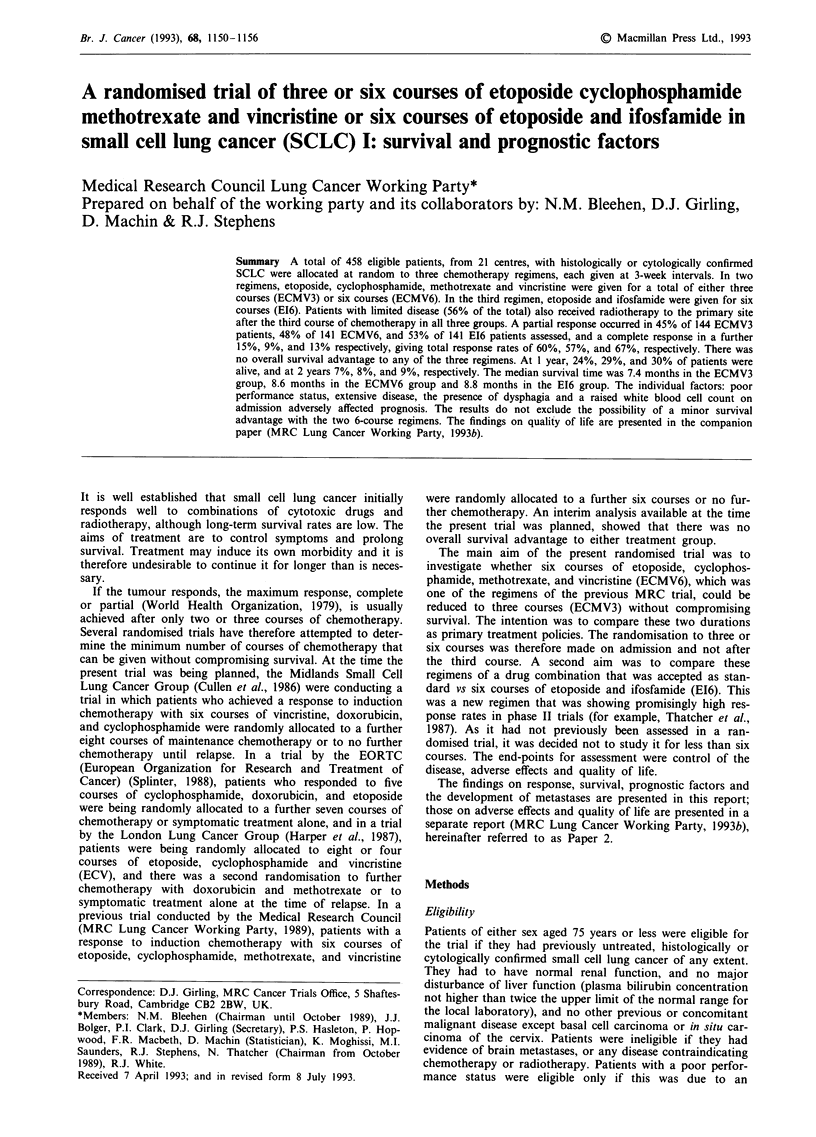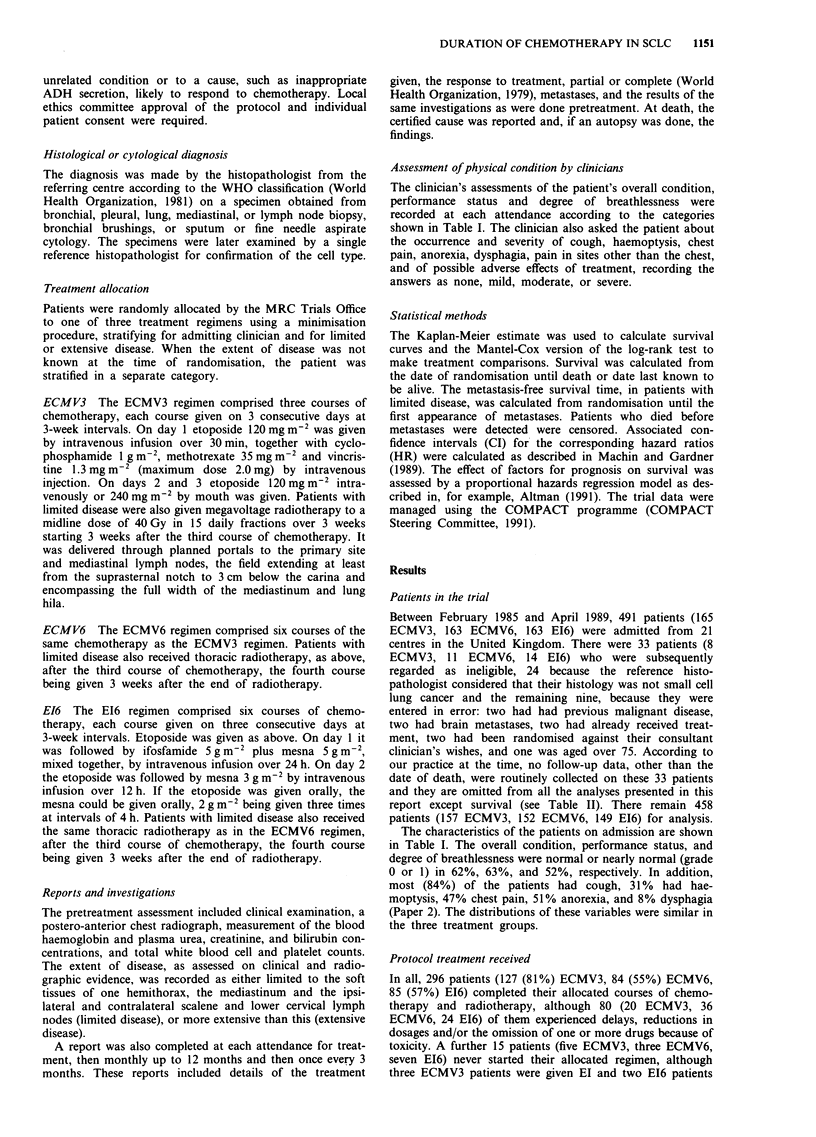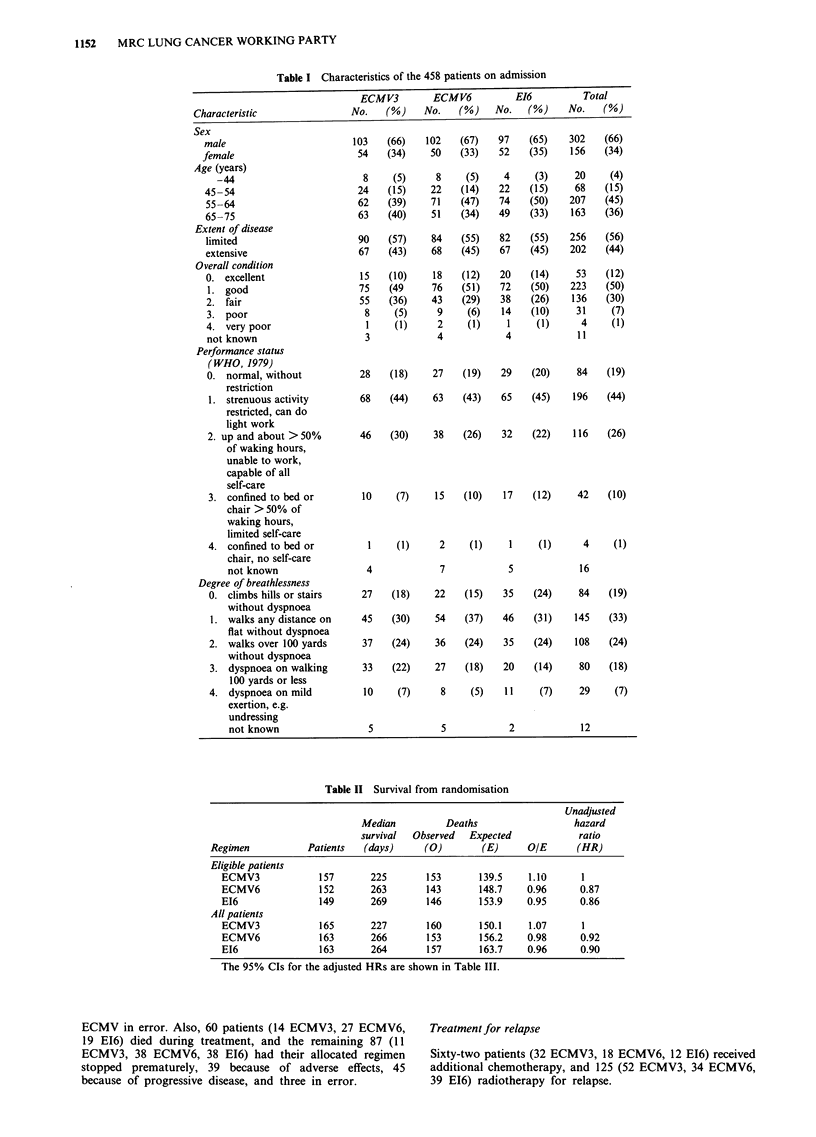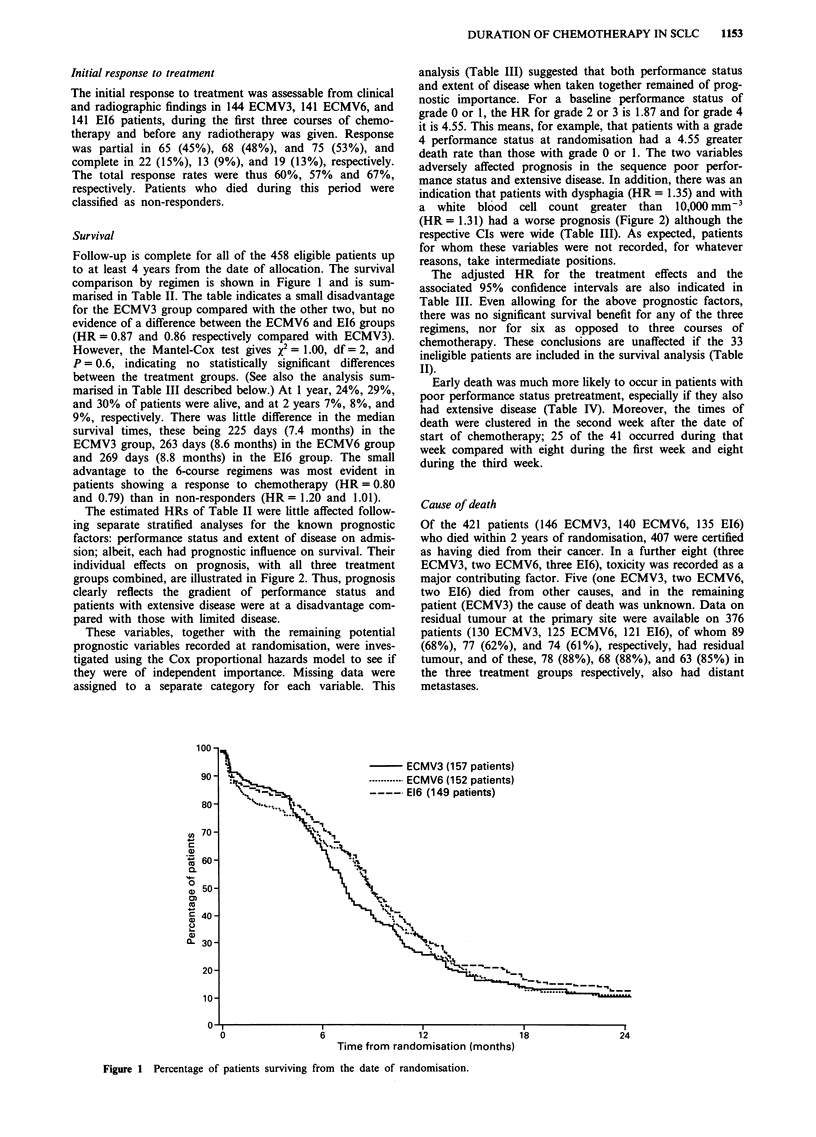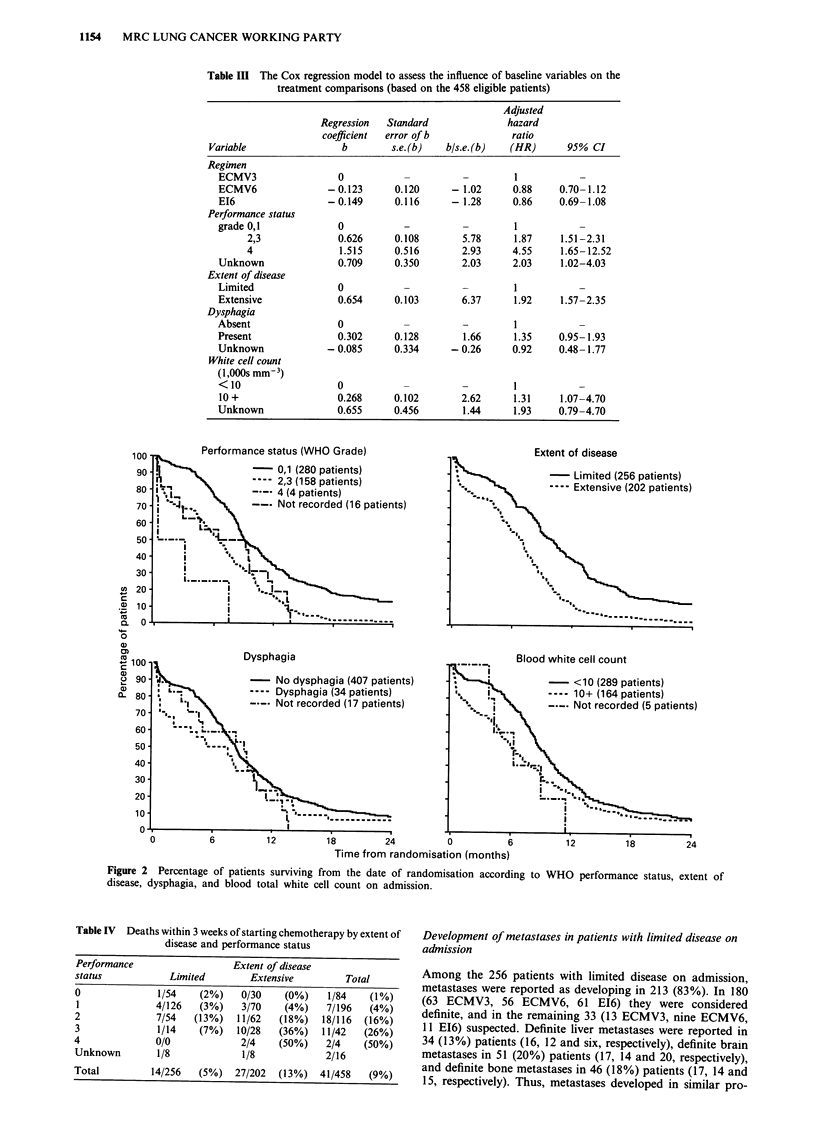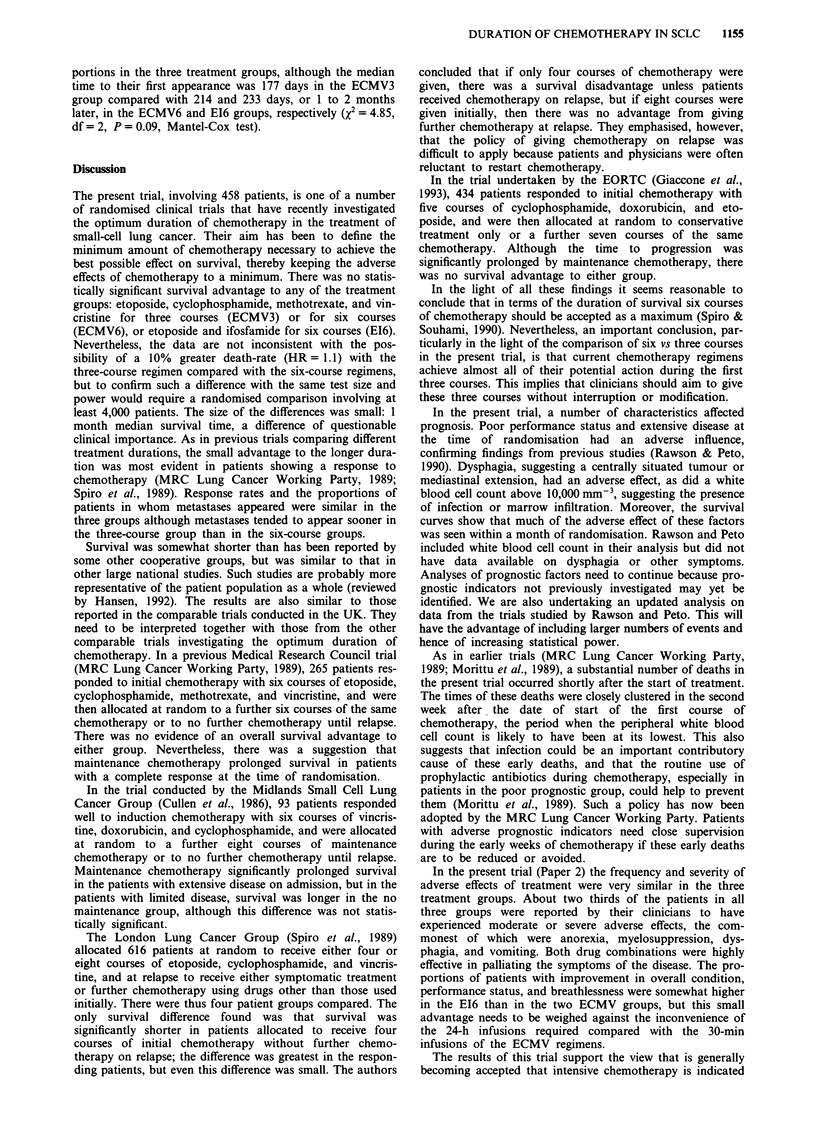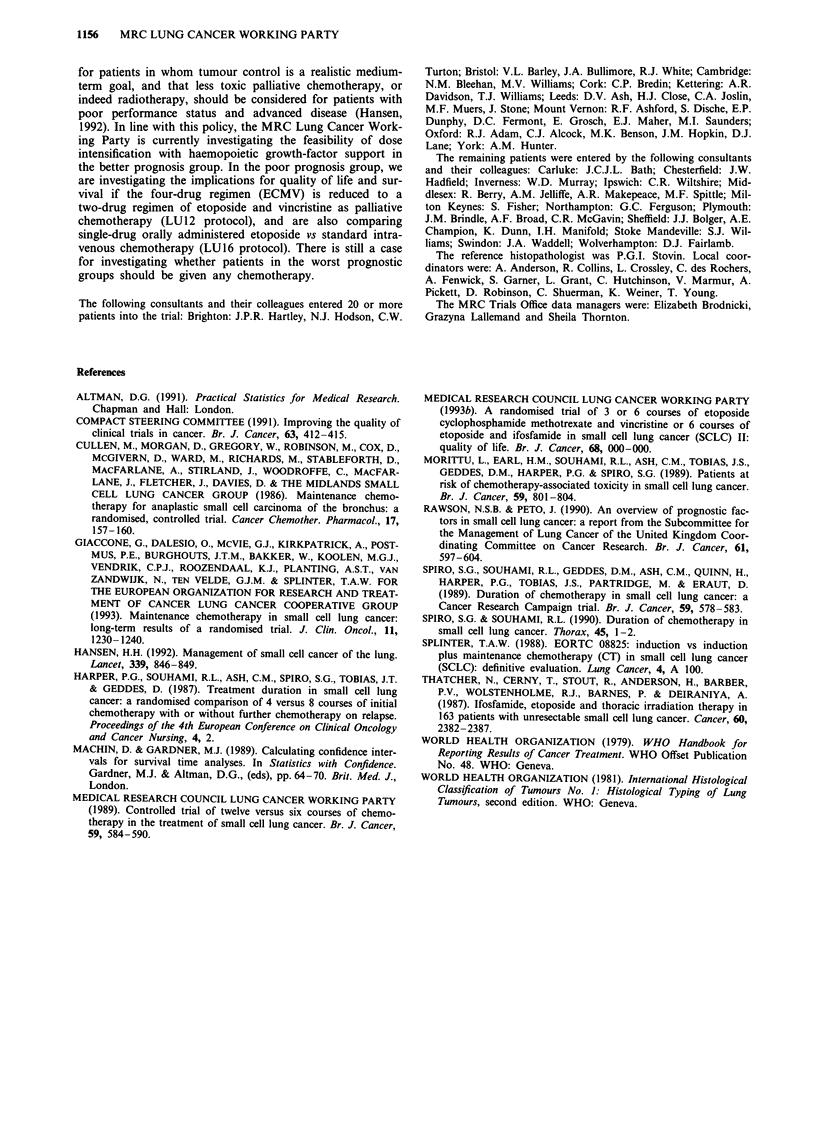# A randomised trial of three or six courses of etoposide cyclophosphamide methotrexate and vincristine or six courses of etoposide and ifosfamide in small cell lung cancer (SCLC). I: Survival and prognostic factors. Medical Research Council Lung Cancer Working Party.

**DOI:** 10.1038/bjc.1993.496

**Published:** 1993-12

**Authors:** N. M. Bleehen, D. J. Girling, D. Machin, R. J. Stephens

## Abstract

A total of 458 eligible patients, from 21 centres, with histologically or cytologically confirmed SCLC were allocated at random to three chemotherapy regimens, each given at 3-week intervals. In two regimens, etoposide, cyclophosphamide, methotrexate and vincristine were given for a total of either three courses (ECMV3) or six courses (ECMV6). In the third regimen, etoposide and ifosfamide were given for six courses (EI6). Patients with limited disease (56% of the total) also received radiotherapy to the primary site after the third course of chemotherapy in all three groups. A partial response occurred in 45% of 144 ECMV3 patients, 48% of 141 ECMV6, and 53% of 141 EI6 patients assessed, and a complete response in a further 15%, 9%, and 13% respectively, giving total response rates of 60%, 57%, and 67%, respectively. There was no overall survival advantage to any of the three regimens. At 1 year, 24%, 29%, and 30% of patients were alive, and at 2 years 7%, 8%, and 9%, respectively. The median survival time was 7.4 months in the ECMV3 group, 8.6 months in the ECMV6 group and 8.8 months in the EI6 group. The individual factors: poor performance status, extensive disease, the presence of dysphagia and a raised white blood cell count on admission adversely affected prognosis. The results do not exclude the possibility of a minor survival advantage with the two 6-course regimens. The findings on quality of life are presented in the companion paper (MRC Lung Cancer Working Party, 1993b).


Br. J. Cancer (1993), 68, 1150–1156 © Macmillan Press Ltd., 1993

### A randomised trial of three or six courses of etoposide cyclophosphamide methotrexate and vincristine or six courses of etoposide and ifosfamide in small cell lung cancer (SCLC) I: survival and prognostic factors

Medical Research Council Lung Cancer Working Party*
Prepared on behalf of the working party and its collaborators by: N.M. Bleehen, D.J. Girling, D. Machin & R.J. Stephens

**Summary** A total of 458 eligible patients, from 21 centres, with histologically or cytologically confirmed SCLC were allocated at random to three chemotherapy regimens, each given at 3-week intervals. In two regimens, etoposide, cyclophosphamide, methotrexate and vincristine were given for a total of either three courses (ECMV3) or six courses (ECMV6). In the third regimen, etoposide and ifosfamide were given for six courses (EI6). Patients with limited disease (56% of the total) also received radiotherapy to the primary site after the third course of chemotherapy in all three groups. A partial response occurred in 45% of 144 ECMV3 patients, 48% of 141 ECMV6, and 53% of 141 EI6 patients assessed, and a complete response in a further 15%, 9%, and 13% respectively, giving total response rates of 60%, 57%, and 67%, respectively. There was no overall survival advantage to any of the three regimens. At 1 year, 24%, 29%, and 30% of patients were alive, and at 2 years 7%, 8%, and 9%, respectively. The median survival time was 7.4 months in the ECMV3 group, 8.6 months in the ECMV6 group and 8.8 months in the EI6 group. The individual factors: poor performance status, extensive disease, the presence of dysphagia and a raised white blood cell count on admission adversely affected prognosis. The results do not exclude the possibility of a minor survival advantage with the two 6-course regimens. The findings on quality of life are presented in the companion paper (MRC Lung Cancer Working Party, 1993*b*).

It is well established that small cell lung cancer initially responds well to combinations of cytotoxic drugs and radiotherapy, although long-term survival rates are low. The aims of treatment are to control symptoms and prolong survival. Treatment may induce its own morbidity and it is therefore undesirable to continue it for longer than is necessary.

If the tumour responds, the maximum response, complete or partial (World Health Organization, 1979), is usually achieved after only two or three courses of chemotherapy. Several randomised trials have therefore attempted to determine the minimum number of courses of chemotherapy that can be given without compromising survival. At the time the present trial was being planned, the Midlands Small Cell Lung Cancer Group (Cullen *et al*., 1986) were conducting a trial in which patients who achieved a response to induction chemotherapy with six courses of vincristine, doxorubicin, and cyclophosphamide were randomly allocated to a further eight courses of maintenance chemotherapy or to no further chemotherapy until relapse. In a trial by the EORTC (European Organization for Research and Treatment of Cancer) (Splinter, 1988), patients who responded to five courses of cyclophosphamide, doxorubicin, and etoposide were being randomly allocated to a further seven courses of chemotherapy or symptomatic treatment alone, and in a trial by the London Lung Cancer Group (Harper *et al*., 1987), patients were being randomly allocated to eight or four courses of etoposide, cyclophosphamide and vincristine (ECV), and there was a second randomisation to further chemotherapy with doxorubicin and methotrexate or to symptomatic treatment alone at the time of relapse. In a previous trial conducted by the Medical Research Council (MRC Lung Cancer Working Party, 1989), patients with a response to induction chemotherapy with six courses of etoposide, cyclophosphamide, methotrexate, and vincristine were randomly allocated to a further six courses or no further chemotherapy. An interim analysis available at the time the present trial was planned, showed that there was no overall survival advantage to either treatment group.

The main aim of the present randomised trial was to investigate whether six courses of etoposide, cyclophosphamide, methotrexate, and vincristine (ECMV6), which was one of the regimens of the previous MRC trial, could be reduced to three courses (ECMV3) without compromising survival. The intention was to compare these two durations as primary treatment policies. The randomisation to three or six courses was therefore made on admission and not after the third course. A second aim was to compare these regimens of a drug combination that was accepted as standard *vs* six courses of etoposide and ifosfamide (EI6). This was a new regimen that was showing promisingly high response rates in phase II trials (for example, Thatcher *et al*., 1987). As it had not previously been assessed in a randomised trial, it was decided not to study it for less than six courses. The end-points for assessment were control of the disease, adverse effects and quality of life.

The findings on response, survival, prognostic factors and the development of metastases are presented in this report; those on adverse effects and quality of life are presented in a separate report (MRC Lung Cancer Working Party, 1993*b*), hereinafter referred to as Paper 2.

### Methods

### *Eligibility*

Patients of either sex aged 75 years or less were eligible for the trial if they had previously untreated, histologically or cytologically confirmed small cell lung cancer of any extent. They had to have normal renal function, and no major disturbance of liver function (plasma bilirubin concentration not higher than twice the upper limit of the normal range for the local laboratory), and no other previous or concomitant malignant disease except basal cell carcinoma or *in situ* carcinoma of the cervix. Patients were ineligible if they had evidence of brain metastases, or any disease contraindicating chemotherapy or radiotherapy. Patients with a poor performance status were eligible only if this was due to an

Correspondence: D.J. Girling, MRC Cancer Trials Office, 5 Shaftesbury Road, Cambridge CB2 2BW, UK.
*Members: N.M. Bleehen (Chairman until October 1989), J.J. Bolger, P.I. Clark, D.J. Girling (Secretary), P.S. Hasleton, P. Hopwood, F.R. Macbeth, D. Machin (Statistician), K. Moghissi, M.I. Saunders, R.J. Stephens, N. Thatcher (Chairman from October 1989), R.J. White.
Received 7 April 1993; and in revised form 8 July 1993.

unrelated condition or to a cause, such as inappropriate ADH secretion, likely to respond to chemotherapy. Local ethics committee approval of the protocol and individual patient consent were required.

*Histological or cytological diagnosis*

The diagnosis was made by the histopathologist from the referring centre according to the WHO classification (World Health Organization, 1981) on a specimen obtained from bronchial, pleural, lung, mediastinal, or lymph node biopsy, bronchial brushings, or sputum or fine needle aspirate cytology. The specimens were later examined by a single reference histopathologist for confirmation of the cell type.

*Treatment allocation*

Patients were randomly allocated by the MRC Trials Office to one of three treatment regimens using a minimisation procedure, stratifying for admitting clinician and for limited or extensive disease. When the extent of disease was not known at the time of randomisation, the patient was stratified in a separate category.

*ECMV3* The ECMV3 regimen comprised three courses of chemotherapy, each course given on 3 consecutive days at 3-week intervals. On day 1 etoposide 120 mg m$^{-2}$ was given by intravenous infusion over 30 min, together with cyclophosphamide 1 g m$^{-2}$, methotrexate 35 mg m$^{-2}$ and vincristine 1.3 mg m$^{-2}$ (maximum dose 2.0 mg) by intravenous injection. On days 2 and 3 etoposide 120 mg m$^{-2}$ intravenously or 240 mg m$^{-2}$ by mouth was given. Patients with limited disease were also given megavoltage radiotherapy to a midline dose of 40 Gy in 15 daily fractions over 3 weeks starting 3 weeks after the third course of chemotherapy. It was delivered through planned portals to the primary site and mediastinal lymph nodes, the field extending at least from the suprasternal notch to 3 cm below the carina and encompassing the full width of the mediastinum and lung hila.

*ECMV6* The ECMV6 regimen comprised six courses of the same chemotherapy as the ECMV3 regimen. Patients with limited disease also received thoracic radiotherapy, as above, after the third course of chemotherapy, the fourth course being given 3 weeks after the end of radiotherapy.

*EI6* The EI6 regimen comprised six courses of chemotherapy, each course given on three consecutive days at 3-week intervals. Etoposide was given as above. On day 1 it was followed by ifosfamide 5 g m$^{-2}$ plus mesna 5 g m$^{-2}$, mixed together, by intravenous infusion over 24 h. On day 2 the etoposide was followed by mesna 3 g m$^{-2}$ by intravenous infusion over 12 h. If the etoposide was given orally, the mesna could be given orally, 2 g m$^{-2}$ being given three times at intervals of 4 h. Patients with limited disease also received the same thoracic radiotherapy as in the ECMV6 regimen, after the third course of chemotherapy, the fourth course being given 3 weeks after the end of radiotherapy.

*Reports and investigations*

The pretreatment assessment included clinical examination, a postero-anterior chest radiograph, measurement of the blood haemoglobin and plasma urea, creatinine, and bilirubin concentrations, and total white blood cell and platelet counts. The extent of disease, as assessed on clinical and radiographic evidence, was recorded as either limited to the soft tissues of one hemithorax, the mediastinum and the ipsilateral and contralateral scalene and lower cervical lymph nodes (limited disease), or more extensive than this (extensive disease).

A report was also completed at each attendance for treatment, then monthly up to 12 months and then once every 3 months. These reports included details of the treatment given, the response to treatment, partial or complete (World Health Organization, 1979), metastases, and the results of the same investigations as were done pretreatment. At death, the certified cause was reported and, if an autopsy was done, the findings.

*Assessment of physical condition by clinicians*

The clinician's assessments of the patient's overall condition, performance status and degree of breathlessness were recorded at each attendance according to the categories shown in Table I. The clinician also asked the patient about the occurrence and severity of cough, haemoptysis, chest pain, anorexia, dysphagia, pain in sites other than the chest, and of possible adverse effects of treatment, recording the answers as none, mild, moderate, or severe.

*Statistical methods*

The Kaplan-Meier estimate was used to calculate survival curves and the Mantel-Cox version of the log-rank test to make treatment comparisons. Survival was calculated from the date of randomisation until death or date last known to be alive. The metastasis-free survival time, in patients with limited disease, was calculated from randomisation until the first appearance of metastases. Patients who died before metastases were detected were censored. Associated confidence intervals (CI) for the corresponding hazard ratios (HR) were calculated as described in Machin and Gardner (1989). The effect of factors for prognosis on survival was assessed by a proportional hazards regression model as described in, for example, Altman (1991). The trial data were managed using the COMPACT programme (COMPACT Steering Committee, 1991).

### Results

*Patients in the trial*

Between February 1985 and April 1989, 491 patients (165 ECMV3, 163 ECMV6, 163 EI6) were admitted from 21 centres in the United Kingdom. There were 33 patients (8 ECMV3, 11 ECMV6, 14 EI6) who were subsequently regarded as ineligible, 24 because the reference histopathologist considered that their histology was not small cell lung cancer and the remaining nine, because they were entered in error: two had had previous malignant disease, two had brain metastases, two had already received treatment, two had been randomised against their consultant clinician's wishes, and one was aged over 75. According to our practice at the time, no follow-up data, other than the date of death, were routinely collected on these 33 patients and they are omitted from all the analyses presented in this report except survival (see Table II). There remain 458 patients (157 ECMV3, 152 ECMV6, 149 EI6) for analysis.

The characteristics of the patients on admission are shown in Table I. The overall condition, performance status, and degree of breathlessness were normal or nearly normal (grade 0 or 1) in 62%, 63%, and 52%, respectively. In addition, most (84%) of the patients had cough, 31% had haemoptysis, 47% chest pain, 51% anorexia, and 8% dysphagia (Paper 2). The distributions of these variables were similar in the three treatment groups.

*Protocol treatment received*

In all, 296 patients (127 (81%) ECMV3, 84 (55%) ECMV6, 85 (57%) EI6) completed their allocated courses of chemotherapy and radiotherapy, although 80 (20 ECMV3, 36 ECMV6, 24 EI6) of them experienced delays, reductions in dosages and/or the omission of one or more drugs because of toxicity. A further 15 patients (five ECMV3, three ECMV6, seven EI6) never started their allocated regimen, although three ECMV3 patients were given EI and two EI6 patients

**Table I** Characteristics of the 458 patients on admission

| Characteristic | *ECMV3* No. | (%) | *ECMV6* No. | (%) | *EI6* No. | (%) | *Total* No. | (%) |
|---|---|---|---|---|---|---|---|---|
| *Sex* | | | | | | | | |
| *male* | 103 | (66) | 102 | (67) | 97 | (65) | 302 | (66) |
| *female* | 54 | (34) | 50 | (33) | 52 | (35) | 156 | (34) |
| *Age* (years) | | | | | | | | |
| -44 | 8 | (5) | 8 | (5) | 4 | (3) | 20 | (4) |
| 45-54 | 24 | (15) | 22 | (14) | 22 | (15) | 68 | (15) |
| 55-64 | 62 | (39) | 71 | (47) | 74 | (50) | 207 | (45) |
| 65-75 | 63 | (40) | 51 | (34) | 49 | (33) | 163 | (36) |
| *Extent of disease* | | | | | | | | |
| limited | 90 | (57) | 84 | (55) | 82 | (55) | 256 | (56) |
| extensive | 67 | (43) | 68 | (45) | 67 | (45) | 202 | (44) |
| *Overall condition* | | | | | | | | |
| 0. excellent | 15 | (10) | 18 | (12) | 20 | (14) | 53 | (12) |
| 1. good | 75 | (49 | 76 | (51) | 72 | (50) | 223 | (50) |
| 2. fair | 55 | (36) | 43 | (29) | 38 | (26) | 136 | (30) |
| 3. poor | 8 | (5) | 9 | (6) | 14 | (10) | 31 | (7) |
| 4. very poor | 1 | (1) | 2 | (1) | 1 | (1) | 4 | (1) |
| not known | 3 | | 4 | | 4 | | 11 | |
| *Performance status (WHO, 1979)* | | | | | | | | |
| 0. normal, without restriction | 28 | (18) | 27 | (19) | 29 | (20) | 84 | (19) |
| 1. strenuous activity restricted, can do light work | 68 | (44) | 63 | (43) | 65 | (45) | 196 | (44) |
| 2. up and about >50% of waking hours, unable to work, capable of all self-care | 46 | (30) | 38 | (26) | 32 | (22) | 116 | (26) |
| 3. confined to bed or chair >50% of waking hours, limited self-care | 10 | (7) | 15 | (10) | 17 | (12) | 42 | (10) |
| 4. confined to bed or chair, no self-care | 1 | (1) | 2 | (1) | 1 | (1) | 4 | (1) |
| not known | 4 | | 7 | | 5 | | 16 | |
| *Degree of breathlessness* | | | | | | | | |
| 0. climbs hills or stairs without dyspnoea | 27 | (18) | 22 | (15) | 35 | (24) | 84 | (19) |
| 1. walks any distance on flat without dyspnoea | 45 | (30) | 54 | (37) | 46 | (31) | 145 | (33) |
| 2. walks over 100 yards without dyspnoea | 37 | (24) | 36 | (24) | 35 | (24) | 108 | (24) |
| 3. dyspnoea on walking 100 yards or less | 33 | (22) | 27 | (18) | 20 | (14) | 80 | (18) |
| 4. dyspnoea on mild exertion, e.g. undressing | 10 | (7) | 8 | (5) | 11 | (7) | 29 | (7) |
| not known | 5 | | 5 | | 2 | | 12 | |

**Table II** Survival from randomisation

| *Regimen* | *Patients* | *Median survival (days)* | *Deaths Observed (O)* | *Deaths Expected (E)* | *O/E* | *Unadjusted hazard ratio (HR)* |
|---|---|---|---|---|---|---|
| *Eligible patients* | | | | | | |
| ECMV3 | 157 | 225 | 153 | 139.5 | 1.10 | 1 |
| ECMV6 | 152 | 263 | 143 | 148.7 | 0.96 | 0.87 |
| EI6 | 149 | 269 | 146 | 153.9 | 0.95 | 0.86 |
| *All patients* | | | | | | |
| ECMV3 | 165 | 227 | 160 | 150.1 | 1.07 | 1 |
| ECMV6 | 163 | 266 | 153 | 156.2 | 0.98 | 0.92 |
| EI6 | 163 | 264 | 157 | 163.7 | 0.96 | 0.90 |

The 95% CIs for the adjusted HRs are shown in Table III.

ECMV in error. Also, 60 patients (14 ECMV3, 27 ECMV6, 19 EI6) died during treatment, and the remaining 87 (11 ECMV3, 38 ECMV6, 38 EI6) had their allocated regimen stopped prematurely, 39 because of adverse effects, 45 because of progressive disease, and three in error.

*Treatment for relapse*

Sixty-two patients (32 ECMV3, 18 ECMV6, 12 EI6) received additional chemotherapy, and 125 (52 ECMV3, 34 ECMV6, 39 EI6) radiotherapy for relapse.

*Initial response to treatment*

The initial response to treatment was assessable from clinical and radiographic findings in 144 ECMV3, 141 ECMV6, and 141 EI6 patients, during the first three courses of chemotherapy and before any radiotherapy was given. Response was partial in 65 (45%), 68 (48%), and 75 (53%), and complete in 22 (15%), 13 (9%), and 19 (13%), respectively. The total response rates were thus 60%, 57% and 67%, respectively. Patients who died during this period were classified as non-responders.

*Survival*

Follow-up is complete for all of the 458 eligible patients up to at least 4 years from the date of allocation. The survival comparison by regimen is shown in Figure 1 and is summarised in Table II. The table indicates a small disadvantage for the ECMV3 group compared with the other two, but no evidence of a difference between the ECMV6 and EI6 groups (HR = 0.87 and 0.86 respectively compared with ECMV3). However, the Mantel-Cox test gives $\chi^2 = 1.00$, df = 2, and $P = 0.6$, indicating no statistically significant differences between the treatment groups. (See also the analysis summarised in Table III described below.) At 1 year, 24%, 29%, and 30% of patients were alive, and at 2 years 7%, 8%, and 9%, respectively. There was little difference in the median survival times, these being 225 days (7.4 months) in the ECMV3 group, 263 days (8.6 months) in the ECMV6 group and 269 days (8.8 months) in the EI6 group. The small advantage to the 6-course regimens was most evident in patients showing a response to chemotherapy (HR = 0.80 and 0.79) than in non-responders (HR = 1.20 and 1.01).

The estimated HRs of Table II were little affected following separate stratified analyses for the known prognostic factors: performance status and extent of disease on admission; albeit, each had prognostic influence on survival. Their individual effects on prognosis, with all three treatment groups combined, are illustrated in Figure 2. Thus, prognosis clearly reflects the gradient of performance status and patients with extensive disease were at a disadvantage compared with those with limited disease.

These variables, together with the remaining potential prognostic variables recorded at randomisation, were investigated using the Cox proportional hazards model to see if they were of independent importance. Missing data were assigned to a separate category for each variable. This analysis (Table III) suggested that both performance status and extent of disease when taken together remained of prognostic importance. For a baseline performance status of grade 0 or 1, the HR for grade 2 or 3 is 1.87 and for grade 4 it is 4.55. This means, for example, that patients with a grade 4 performance status at randomisation had a 4.55 greater death rate than those with grade 0 or 1. The two variables adversely affected prognosis in the sequence poor performance status and extensive disease. In addition, there was an indication that patients with dysphagia (HR = 1.35) and with a white blood cell count greater than 10,000 $mm^{-3}$ (HR = 1.31) had a worse prognosis (Figure 2) although the respective CIs were wide (Table III). As expected, patients for whom these variables were not recorded, for whatever reasons, take intermediate positions.

The adjusted HR for the treatment effects and the associated 95% confidence intervals are also indicated in Table III. Even allowing for the above prognostic factors, there was no significant survival benefit for any of the three regimens, nor for six as opposed to three courses of chemotherapy. These conclusions are unaffected if the 33 ineligible patients are included in the survival analysis (Table II).

Early death was much more likely to occur in patients with poor performance status pretreatment, especially if they also had extensive disease (Table IV). Moreover, the times of death were clustered in the second week after the date of start of chemotherapy; 25 of the 41 occurred during that week compared with eight during the first week and eight during the third week.

*Cause of death*

Of the 421 patients (146 ECMV3, 140 ECMV6, 135 EI6) who died within 2 years of randomisation, 407 were certified as having died from their cancer. In a further eight (three ECMV3, two ECMV6, three EI6), toxicity was recorded as a major contributing factor. Five (one ECMV3, two ECMV6, two EI6) died from other causes, and in the remaining patient (ECMV3) the cause of death was unknown. Data on residual tumour at the primary site were available on 376 patients (130 ECMV3, 125 ECMV6, 121 EI6), of whom 89 (68%), 77 (62%), and 74 (61%), respectively, had residual tumour, and of these, 78 (88%), 68 (88%), and 63 (85%) in the three treatment groups respectively, also had distant metastases.

**Figure 1** Percentage of patients surviving from the date of randomisation.

**Table III** The Cox regression model to assess the influence of baseline variables on the treatment comparisons (based on the 458 eligible patients)

| *Variable* | *Regression coefficient b* | *Standard error of b s.e.(b)* | *b/s.e.(b)* | *Adjusted hazard ratio (HR)* | *95% CI* |
|---|---|---|---|---|---|
| *Regimen* | | | | | |
| ECMV3 | 0 | – | – | 1 | – |
| ECMV6 | – 0.123 | 0.120 | – 1.02 | 0.88 | 0.70–1.12 |
| EI6 | – 0.149 | 0.116 | – 1.28 | 0.86 | 0.69–1.08 |
| *Performance status* | | | | | |
| grade 0,1 | 0 | – | – | 1 | – |
| 2,3 | 0.626 | 0.108 | 5.78 | 1.87 | 1.51–2.31 |
| 4 | 1.515 | 0.516 | 2.93 | 4.55 | 1.65–12.52 |
| Unknown | 0.709 | 0.350 | 2.03 | 2.03 | 1.02–4.03 |
| *Extent of disease* | | | | | |
| Limited | 0 | – | – | 1 | – |
| Extensive | 0.654 | 0.103 | 6.37 | 1.92 | 1.57–2.35 |
| *Dysphagia* | | | | | |
| Absent | 0 | – | – | 1 | – |
| Present | 0.302 | 0.128 | 1.66 | 1.35 | 0.95–1.93 |
| Unknown | – 0.085 | 0.334 | – 0.26 | 0.92 | 0.48–1.77 |
| *White cell count* (1,000s mm$^{-3}$) | | | | | |
| <10 | 0 | – | – | 1 | – |
| 10 + | 0.268 | 0.102 | 2.62 | 1.31 | 1.07–4.70 |
| Unknown | 0.655 | 0.456 | 1.44 | 1.93 | 0.79–4.70 |

**Figure 2** Percentage of patients surviving from the date of randomisation according to WHO performance status, extent of disease, dysphagia, and blood total white cell count on admission.

**Table IV** Deaths within 3 weeks of starting chemotherapy by extent of disease and performance status

| *Performance status* | *Extent of disease* *Limited* | | *Extensive* | | *Total* | |
|---|---|---|---|---|---|---|
| 0 | 1/54 | (2%) | 0/30 | (0%) | 1/84 | (1%) |
| 1 | 4/126 | (3%) | 3/70 | (4%) | 7/196 | (4%) |
| 2 | 7/54 | (13%) | 11/62 | (18%) | 18/116 | (16%) |
| 3 | 1/14 | (7%) | 10/28 | (36%) | 11/42 | (26%) |
| 4 | 0/0 | | 2/4 | (50%) | 2/4 | (50%) |
| Unknown | 1/8 | | 1/8 | | 2/16 | |
| Total | 14/256 | (5%) | 27/202 | (13%) | 41/458 | (9%) |

*Development of metastases in patients with limited disease on admission*

Among the 256 patients with limited disease on admission, metastases were reported as developing in 213 (83%). In 180 (63 ECMV3, 56 ECMV6, 61 EI6) they were considered definite, and in the remaining 33 (13 ECMV3, nine ECMV6, 11 EI6) suspected. Definite liver metastases were reported in 34 (13%) patients (16, 12 and six, respectively), definite brain metastases in 51 (20%) patients (17, 14 and 20, respectively), and definite bone metastases in 46 (18%) patients (17, 14 and 15, respectively). Thus, metastases developed in similar proportions in the three treatment groups, although the median time to their first appearance was 177 days in the ECMV3 group compared with 214 and 233 days, or 1 to 2 months later, in the ECMV6 and EI6 groups, respectively ($\chi^2 = 4.85$, df = 2, $P = 0.09$, Mantel-Cox test).

### Discussion

The present trial, involving 458 patients, is one of a number of randomised clinical trials that have recently investigated the optimum duration of chemotherapy in the treatment of small-cell lung cancer. Their aim has been to define the minimum amount of chemotherapy necessary to achieve the best possible effect on survival, thereby keeping the adverse effects of chemotherapy to a minimum. There was no statistically significant survival advantage to any of the treatment groups: etoposide, cyclophosphamide, methotrexate, and vincristine for three courses (ECMV3) or for six courses (ECMV6), or etoposide and ifosfamide for six courses (EI6). Nevertheless, the data are not inconsistent with the possibility of a 10% greater death-rate (HR = 1.1) with the three-course regimen compared with the six-course regimens, but to confirm such a difference with the same test size and power would require a randomised comparison involving at least 4,000 patients. The size of the differences was small: 1 month median survival time, a difference of questionable clinical importance. As in previous trials comparing different treatment durations, the small advantage to the longer duration was most evident in patients showing a response to chemotherapy (MRC Lung Cancer Working Party, 1989; Spiro *et al.*, 1989). Response rates and the proportions of patients in whom metastases appeared were similar in the three groups although metastases tended to appear sooner in the three-course group than in the six-course groups.

Survival was somewhat shorter than has been reported by some other cooperative groups, but was similar to that in other large national studies. Such studies are probably more representative of the patient population as a whole (reviewed by Hansen, 1992). The results are also similar to those reported in the comparable trials conducted in the UK. They need to be interpreted together with those from the other comparable trials investigating the optimum duration of chemotherapy. In a previous Medical Research Council trial (MRC Lung Cancer Working Party, 1989), 265 patients responded to initial chemotherapy with six courses of etoposide, cyclophosphamide, methotrexate, and vincristine, and were then allocated at random to a further six courses of the same chemotherapy or to no further chemotherapy until relapse. There was no evidence of an overall survival advantage to either group. Nevertheless, there was a suggestion that maintenance chemotherapy prolonged survival in patients with a complete response at the time of randomisation.

In the trial conducted by the Midlands Small Cell Lung Cancer Group (Cullen *et al.*, 1986), 93 patients responded well to induction chemotherapy with six courses of vincristine, doxorubicin, and cyclophosphamide, and were allocated at random to a further eight courses of maintenance chemotherapy or to no further chemotherapy until relapse. Maintenance chemotherapy significantly prolonged survival in the patients with extensive disease on admission, but in the patients with limited disease, survival was longer in the no maintenance group, although this difference was not statistically significant.

The London Lung Cancer Group (Spiro *et al.*, 1989) allocated 616 patients at random to receive either four or eight courses of etoposide, cyclophosphamide, and vincristine, and at relapse to receive either symptomatic treatment or further chemotherapy using drugs other than those used initially. There were thus four patient groups compared. The only survival difference found was that survival was significantly shorter in patients allocated to receive four courses of initial chemotherapy without further chemotherapy on relapse; the difference was greatest in the responding patients, but even this difference was small. The authors concluded that if only four courses of chemotherapy were given, there was a survival disadvantage unless patients received chemotherapy on relapse, but if eight courses were given initially, then there was no advantage from giving further chemotherapy at relapse. They emphasised, however, that the policy of giving chemotherapy on relapse was difficult to apply because patients and physicians were often reluctant to restart chemotherapy.

In the trial undertaken by the EORTC (Giaccone *et al.*, 1993), 434 patients responded to initial chemotherapy with five courses of cyclophosphamide, doxorubicin, and etoposide, and were then allocated at random to conservative treatment only or a further seven courses of the same chemotherapy. Although the time to progression was significantly prolonged by maintenance chemotherapy, there was no survival advantage to either group.

In the light of all these findings it seems reasonable to conclude that in terms of the duration of survival six courses of chemotherapy should be accepted as a maximum (Spiro & Souhami, 1990). Nevertheless, an important conclusion, particularly in the light of the comparison of six *vs* three courses in the present trial, is that current chemotherapy regimens achieve almost all of their potential action during the first three courses. This implies that clinicians should aim to give these three courses without interruption or modification.

In the present trial, a number of characteristics affected prognosis. Poor performance status and extensive disease at the time of randomisation had an adverse influence, confirming findings from previous studies (Rawson & Peto, 1990). Dysphagia, suggesting a centrally situated tumour or mediastinal extension, had an adverse effect, as did a white blood cell count above 10,000 $mm^{-3}$, suggesting the presence of infection or marrow infiltration. Moreover, the survival curves show that much of the adverse effect of these factors was seen within a month of randomisation. Rawson and Peto included white blood cell count in their analysis but did not have data available on dysphagia or other symptoms. Analyses of prognostic factors need to continue because prognostic indicators not previously investigated may yet be identified. We are also undertaking an updated analysis on data from the trials studied by Rawson and Peto. This will have the advantage of including larger numbers of events and hence of increasing statistical power.

As in earlier trials (MRC Lung Cancer Working Party, 1989; Morittu *et al.*, 1989), a substantial number of deaths in the present trial occurred shortly after the start of treatment. The times of these deaths were closely clustered in the second week after the date of start of the first course of chemotherapy, the period when the peripheral white blood cell count is likely to have been at its lowest. This also suggests that infection could be an important contributory cause of these early deaths, and that the routine use of prophylactic antibiotics during chemotherapy, especially in patients in the poor prognostic group, could help to prevent them (Morittu *et al.*, 1989). Such a policy has now been adopted by the MRC Lung Cancer Working Party. Patients with adverse prognostic indicators need close supervision during the early weeks of chemotherapy if these early deaths are to be reduced or avoided.

In the present trial (Paper 2) the frequency and severity of adverse effects of treatment were very similar in the three treatment groups. About two thirds of the patients in all three groups were reported by their clinicians to have experienced moderate or severe adverse effects, the commonest of which were anorexia, myelosuppression, dysphagia, and vomiting. Both drug combinations were highly effective in palliating the symptoms of the disease. The proportions of patients with improvement in overall condition, performance status, and breathlessness were somewhat higher in the EI6 than in the two ECMV groups, but this small advantage needs to be weighed against the inconvenience of the 24-h infusions required compared with the 30-min infusions of the ECMV regimens.

The results of this trial support the view that is generally becoming accepted that intensive chemotherapy is indicated for patients in whom tumour control is a realistic medium-term goal, and that less toxic palliative chemotherapy, or indeed radiotherapy, should be considered for patients with poor performance status and advanced disease (Hansen, 1992). In line with this policy, the MRC Lung Cancer Working Party is currently investigating the feasibility of dose intensification with haemopoietic growth-factor support in the better prognosis group. In the poor prognosis group, we are investigating the implications for quality of life and survival if the four-drug regimen (ECMV) is reduced to a two-drug regimen of etoposide and vincristine as palliative chemotherapy (LU12 protocol), and are also comparing single-drug orally administered etoposide *vs* standard intravenous chemotherapy (LU16 protocol). There is still a case for investigating whether patients in the worst prognostic groups should be given any chemotherapy.

The following consultants and their colleagues entered 20 or more patients into the trial: Brighton: J.P.R. Hartley, N.J. Hodson, C.W. Turton; Bristol: V.L. Barley, J.A. Bullimore, R.J. White; Cambridge: N.M. Bleehan, M.V. Williams; Cork: C.P. Bredin; Kettering: A.R. Davidson, T.J. Williams; Leeds: D.V. Ash, H.J. Close, C.A. Joslin, M.F. Muers, J. Stone; Mount Vernon: R.F. Ashford, S. Dische, E.P. Dunphy, D.C. Fermont, E. Grosch, E.J. Maher, M.I. Saunders; Oxford: R.J. Adam, C.J. Alcock, M.K. Benson, J.M. Hopkin, D.J. Lane; York: A.M. Hunter.

The remaining patients were entered by the following consultants and their colleagues: Carluke: J.C.J.L. Bath; Chesterfield: J.W. Hadfield; Inverness: W.D. Murray; Ipswich: C.R. Wiltshire; Middlesex: R. Berry, A.M. Jelliffe, A.R. Makepeace, M.F. Spittle; Milton Keynes: S. Fisher; Northampton: G.C. Ferguson; Plymouth: J.M. Brindle, A.F. Broad, C.R. McGavin; Sheffield: J.J. Bolger, A.E. Champion, K. Dunn, I.H. Manifold; Stoke Mandeville: S.J. Willliams; Swindon: J.A. Waddell; Wolverhampton: D.J. Fairlamb.

The reference histopathologist was P.G.I. Stovin. Local coordinators were: A. Anderson, R. Collins, L. Crossley, C. des Rochers, A. Fenwick, S. Garner, L. Grant, C. Hutchinson, V. Marmur, A. Pickett, D. Robinson, C. Shuerman, K. Weiner, T. Young.

The MRC Trials Office data managers were: Elizabeth Brodnicki, Grazyna Lallemand and Sheila Thornton.

### References

ALTMAN, D.G. (1991). *Practical Statistics for Medical Research.* Chapman and Hall: London.

COMPACT STEERING COMMITTEE (1991). Improving the quality of clinical trials in cancer. *Br. J. Cancer*, **63**, 412–415.

CULLEN, M., MORGAN, D., GREGORY, W., ROBINSON, M., COX, D., MCGIVERN, D., WARD, M., RICHARDS, M., STABLEFORTH, D., MACFARLANE, A., STIRLAND, J., WOODROFFE, C., MACFARLANE, J., FLETCHER, J., DAVIES, D. & THE MIDLANDS SMALL CELL LUNG CANCER GROUP (1986). Maintenance chemotherapy for anaplastic small cell carcinoma of the bronchus: a randomised, controlled trial. *Cancer Chemother. Pharmacol.*, **17**, 157–160.

GIACCONE, G., DALESIO, O., MCVIE, G.J., KIRKPATRICK, A., POSTMUS, P.E., BURGHOUTS, J.T.M., BAKKER, W., KOOLEN, M.G.J., VENDRIK, C.P.J., ROOZENDAAL, K.J., PLANTING, A.S.T., VAN ZANDWIJK, N., TEN VELDE, G.J.M. & SPLINTER, T.A.W. FOR THE EUROPEAN ORGANIZATION FOR RESEARCH AND TREATMENT OF CANCER LUNG CANCER COOPERATIVE GROUP (1993). Maintenance chemotherapy in small cell lung cancer: long-term results of a randomised trial. *J. Clin. Oncol.*, **11**, 1230–1240.

HANSEN, H.H. (1992). Management of small cell cancer of the lung. *Lancet*, **339**, 846–849.

HARPER, P.G., SOUHAMI, R.L., ASH, C.M., SPIRO, S.G., TOBIAS, J.T. & GEDDES, D. (1987). Treatment duration in small cell lung cancer: a randomised comparison of 4 versus 8 courses of initial chemotherapy with or without further chemotherapy on relapse. *Proceedings of the 4th European Conference on Clinical Oncology and Cancer Nursing*, **4**, 2.

MACHIN, D. & GARDNER, M.J. (1989). Calculating confidence intervals for survival time analyses. In *Statistics with Confidence.* Gardner, M.J. & Altman, D.G., (eds), pp. 64–70. *Brit. Med. J.*, London.

MEDICAL RESEARCH COUNCIL LUNG CANCER WORKING PARTY (1989). Controlled trial of twelve versus six courses of chemotherapy in the treatment of small cell lung cancer. *Br. J. Cancer*, **59**, 584–590.

MEDICAL RESEARCH COUNCIL LUNG CANCER WORKING PARTY (1993*b*). A randomised trial of 3 or 6 courses of etoposide cyclophosphamide methotrexate and vincristine or 6 courses of etoposide and ifosfamide in small cell lung cancer (SCLC) II: quality of life. *Br. J. Cancer*, **68**, 000–000.

MORITTU, L., EARL, H.M., SOUHAMI, R.L., ASH, C.M., TOBIAS, J.S., GEDDES, D.M., HARPER, P.G. & SPIRO, S.G. (1989). Patients at risk of chemotherapy-associated toxicity in small cell lung cancer. *Br. J. Cancer*, **59**, 801–804.

RAWSON, N.S.B. & PETO, J. (1990). An overview of prognostic factors in small cell lung cancer: a report from the Subcommittee for the Management of Lung Cancer of the United Kingdom Coordinating Committee on Cancer Research. *Br. J. Cancer*, **61**, 597–604.

SPIRO, S.G., SOUHAMI, R.L., GEDDES, D.M., ASH, C.M., QUINN, H., HARPER, P.G., TOBIAS, J.S., PARTRIDGE, M. & ERAUT, D. (1989). Duration of chemotherapy in small cell lung cancer: a Cancer Research Campaign trial. *Br. J. Cancer*, **59**, 578–583.

SPIRO, S.G. & SOUHAMI, R.L. (1990). Duration of chemotherapy in small cell lung cancer. *Thorax*, **45**, 1–2.

SPLINTER, T.A.W. (1988). EORTC 08825: induction vs induction plus maintenance chemotherapy (CT) in small cell lung cancer (SCLC): definitive evaluation. *Lung Cancer*, **4**, A 100.

THATCHER, N., CERNY, T., STOUT, R., ANDERSON, H., BARBER, P.V., WOLSTENHOLME, R.J., BARNES, P. & DEIRANIYA, A. (1987). Ifosfamide, etoposide and thoracic irradiation therapy in 163 patients with unresectable small cell lung cancer. *Cancer*, **60**, 2382–2387.

WORLD HEALTH ORGANIZATION (1979). *WHO Handbook for Reporting Results of Cancer Treatment.* WHO Offset Publication No. 48. WHO: Geneva.

WORLD HEALTH ORGANIZATION (1981). *International Histological Classification of Tumours No. 1: Histological Typing of Lung Tumours*, second edition. WHO: Geneva.